# Validation of Enhancing Effects of Curcumin on Radiotherapy with F98/*FGT* Glioblastoma-Bearing Rat Model

**DOI:** 10.3390/ijms21124385

**Published:** 2020-06-19

**Authors:** Wei-Hsun Wang, Chao-Yu Shen, Yi-Chun Chien, Wen-Shin Chang, Chia-Wen Tsai, Yi-Hsien Lin, Jeng-Jong Hwang

**Affiliations:** 1 Department of Orthopedic Surgery, Changhua Christian Hospital, Changhua 500, Taiwan; cmch10011@gmail.com; 2 Department of Medical Imaging and Radiology, Shu-Zen Junior College of Medicine and Management, Kaohsiung 821, Taiwan; 3 Institute of Medicine, Chung Shan Medical University, Taichung 402, Taiwan; cs1840@csmu.edu.tw or; 4 Department of Medical Imaging, Chung Shan Medical University Hospital, Taichung 402, Taiwan; 5 Department of Medical Imaging and Radiological Sciences, I-Shou University, Jiaosu Village, Kaohsiung 824, Taiwan; chienyc@isu.edu.tw; 6 School of Medicine, I-Shou University, Jiaosu Village, Kaohsiung 824, Taiwan; 7 Terry Fox Cancer Research Laboratory, Department of Medical Research, China Medical University Hospital, Taichung 402, Taiwan; halittlemelon@hotmail.com (W.-S.C.); wenwen816@gmail.com (C.-W.T.); 8 Division of Radiotherapy, Cheng Hsin General Hospital, No. 45, Cheng Hsin St, Beitou, Taipei 112, Taiwan; 9 School of Medicine, National Yang-Ming University, Taipei 112, Taiwan; 10 Department of Medical Imaging, Chung Shan Medical University Hospital, No. 110, Sec. 1, Jianguo North Road, Taichung 402, Taiwan; 11 Department of Medical Imaging and Radiological Sciences, Chung Shan Medical University, Taichung 112, Taiwan

**Keywords:** curcumin, F98/*FGT*, glioblastoma, radiosensitizer

## Abstract

Glioblastoma, the most common and aggressive brain tumor with low survival rate, is difficult to be cured by neurosurgery or radiotherapy. Mounting evidence has reported the anti-inflammatory and anticancer effects of curcumin on several types of cancer in preclinical studies and clinical trials. To our knowledge, there is no platform or system that could be used to effectively and real-timely evaluate the therapeutic efficacy of curcumin for glioblastoma multiforme (GBM). In this study, we constructed a lentivirus vector with triple-reporter genes (*Fluc*/*GFP*/*tk*) and transduced into rat F98 glioblastoma cells to establish an orthotopic F98/*FGT* glioma-bearing rat model. In the model, the therapeutic efficacies for curcumin alone, radiation alone, and their combination were evaluated via noninvasive bioluminescent imaging and overall survival measurements. At the cell level, curcumin is capable of causing a G2/M cell cycle arrest and sensitizing the F98 cells to radiation. In animal model, curcumin synergistically enhances the effects of radiotherapy on suppressing the growth of both transplanted glioma cells and in situ brain tumors, and extending the overall survival periods longer than those of curcumin alone and radiation alone treatments. In conclusion, we have demonstrated that curcumin may serve as a novel radiosensitizer to combine with radiotherapy using the triple-reporter F98/*FGT* animal model for effective and simultaneous evaluation of therapeutic efficacy.

## 1. Introduction

Glioblastoma or glioblastoma multiform (GBM) is the most frequent, aggressive, and high-grade (grade IV) primary tumor in the central nervous system that occurs in the brain. In spite of those remarkable progressions in neurosurgery, drug developments (gene-targeted therapy, stem-cell-based therapy, immuno-therapy, nanotechnology-based therapy), and advanced radiation technology have been made in the recent decades, the treatment outcomes of various types of cancer, including GBM, are still an important concern and far from satisfied [1,2,3,4,5]. Currently, the standard treatments for GBM are a combination of surgery, radiotherapy, and chemotherapy. GBM is significantly different from other tumors. The present-day neurosurgical treatment for GBM should pay more attention on removing the maximum amount of malignant tumor tissue without damaging the critical areas of the normal brain which when resected would result in a serious postoperative neurological defect. However, the median span of survival in patients following diagnosis with treatment is about 14—15 months, and the 2-year survival rate is less than 25%. The survival of those patients without treatment is usually only a few months [6,7,8,9]. Since the survival outcomes are so far from satisfaction, the clinical and translational scientists are searching for more practicable combinations of chemotherapy or chemoradiotherapy for GBM [9].

As far as radiation therapy is concerned, it is currently the noninvasive and most effective treatment for GBM. With the advancement of technology in radiation therapy, lots of radiotherapeutic models have been developed rapidly to more accurately deliver the radiation doses on the tumor area and avoid causing the damage to the normal tissue areas [10]. However, it is usually found that for those brain tumors with high radiation resistance, there is an extremely high rate of recurrence postradiotherapy [11]. Studies have shown that the characteristics of hypoxia in brain tumors are the main cause of high radiation resistance [12]. In addition, several intrinsic and acquired factors have been reported to contribute to the GBM resistance to chemotherapy and radiotherapy [13,14,15]. Therefore, the radiosensitizer(s) which could significantly enhance the therapeutic efficacy of radiation treatment while causing acceptable normal tissue damage will be a medical breakthrough in the treatment of GBM.

In recent years, herbal and natural compounds together with their derivatives have attracted much attention as promising therapeutic agents and their availability and efficacy in treating all types of cancer, including GBM, are under examination. Curcumin is an herbal natural polyphenol extracted from turmeric (root of the rhizome *Curcuma longa*) [16,17], which possesses multiple biological activities, including antioxidant [18], anti-inflammatory [18], cardioprotective [19], neuroprotective [20], as well as antitumor capacities [21]. Noticeably, the lipophilic properties and specific ability to cross into blood–brain barrier (BBB) make curcumin an efficient agent examined for its preventive and therapeutic efficacy in CNS-related diseases, such as Alzheimer’s disease [22], Parkinson disease [23], and GBM [24].

In spite of the beneficial effects of curcumin, the low bioavailability obstructs its potential utilization in medicinal and pharmaceutical practices. To overcome this disadvantage, scientists have developed several curcumin derivatives, such as demethoxycurcumin (DMC), tetrahydrocurcumin (THC), turmerones, and bisdemethoxycurcumin and have examined their improvements in bioavailability and biological activities [25]. On the other hand, the researches and developments of molecular imaging technology have triggered lots of significant progresses in both molecular biology and medical practices. We believe that the therapeutical bioavailability and efficacy of curcumin can be followed up with powerful molecular imaging systems.

The purpose of this study is to establish a novel brain tumor-bearing animal model for effective and simultaneous evaluation of the therapeutic efficacy of curcumin combined with radiotherapy. There are three main molecular imaging methods: optical, MRI, and nuclear medicine modalities. These noninvasive imaging techniques provide the convenience and accuracy of preclinical animal studies. These modalities allow for increased repetition and frequency of observation, especially for therapeutic use [26,27,28]. In this study, we designed and constructed a lentivirus (*pFu-FGT*) with three reporter genes, *green fluorescent protein* (*GFP*), *luciferase* (*luc*), and *herpes simplex virus type 1-thymidine kinase* (*HSV1-tk*), transfected it into the glioma cell line F98, and evaluated the antitumor capacities of curcumin alone, radiation alone, and curcumin plus radiation at cell and animal levels with a multimodality molecular imaging methodology. In detail, a rat model with in situ brain tumor cells containing the three reporter genes mentioned above was established, and the therapeutic effects of curcumin combined with radiotherapy were investigated by bioluminescence and magnetic resonance imaging. At cell level, the cytotoxicity of curcumin alone on glioblastoma F98 cells was validated, and the mechanisms of curcumin-induced cell cycle alterations contributing to radiation sensitivity of brain tumors to radiation therapy was revealed.

## 2. Results

### 2.1. The Gene Map of the Lentiviral-Based Trifusion Reporter Vector and Survival Curves of F98 Glioblastoma Cells with Curcumin Alone and Radiation Alone

**Figure 1:**
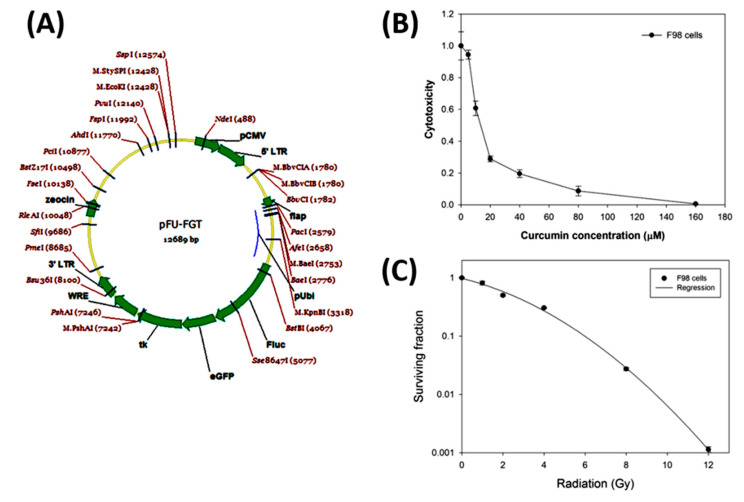
Lentiviral-based trifusion reporter vector, cytotoxicity of curcumin on F98 glioblastoma cells, and radiation survival curve of F98 glioblastoma cells. (**A**) The gene map of constructed lentiviral-based trifusion reporter vector. *FGT*: F stands for *Fluc*: firefly luciferase; G stands for *eGFP*: enhanced green fluorescent protein; T stands for *tk*: thymidine kinase. pUbi: ubiquitin promoter; LTR: long terminal repeat. (**B**) F98 cells were treated with various concentrations of curcumin (0–160 µM) for 24 h and determined with MTT assay. IC_50_ is about 15 µM. (**C**) F98 cells were irradiated with 0–12 Gy of X-ray, and surviving fractions were assayed by colony formation. D_10_ is about 6 Gy.

The gene map of lentiviral-based trifusion vector is shown in Figure 1A. The cytotoxic effect of curcumin on F98 glioblastoma cells was evaluated with MTT assay. The F98 glioblastoma cells were treated with indicated doses of curcumin for 24 h, and the IC_50_ fell between the concentrations of 10–15 μM. At the concentration of higher than 80 μM, the cell survival rate was less than 10% (Figure 1B). The survival fractions of F98 glioblastoma cells after radiation were examined with colony formation assay. The F98 glioblastoma cells were irradiated with indicated radiation doses, and the survival fractions for those cells at 14 days after 1, 2, 4, 8, and 12 Gy irradiation were 0.81, 0.50, 0.31, 0.03, and 0.001, respectively (Figure 1C).

### 2.2. Cell Cycle Alterations after Curcumin Treatment

**Figure 2:**
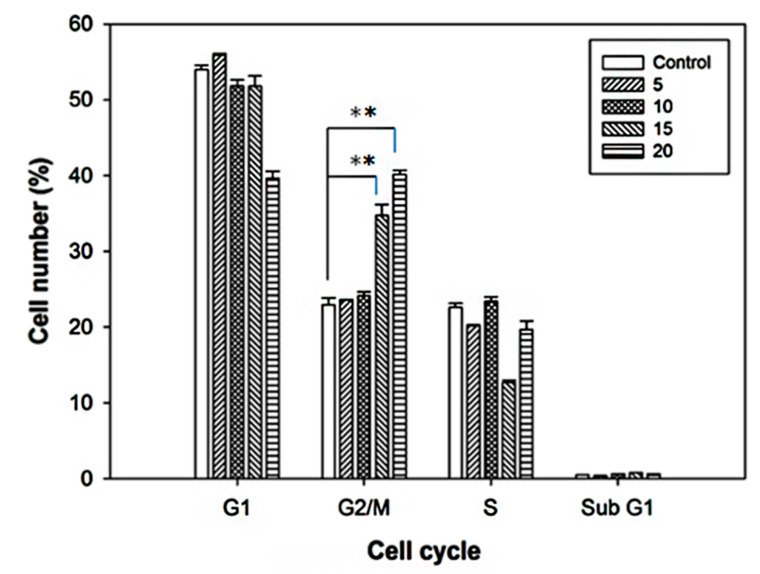
Cell cycle analysis of F98 cells treated with curcumin. F98 cells are treated with various concentrations of curcumin (0, 5, 10, 15, and 20 µM) for 24 h, and then assayed with flow cytometer. The cells were arrested significantly at G2/M phases. ** *p* < 0.01.

The results showed that after treatment of tumor cells with 15 and 20 μM curcumin for 24 h, the ratio of G2/M was significantly increased compared with the control group (Figure 2). It can be inferred that curcumin has an effect of increasing the sensitivity of F98 cells to radiation. Under the microscope, the morphology of the F98 cells treated with 20 μM curcumin has dramatically changed (data not shown).

### 2.3. Transfection of pFu-FGT Lentivirus into F98 Cell Line to Form Unsorted Primary F98/FGT Cells

**Figure 3:**
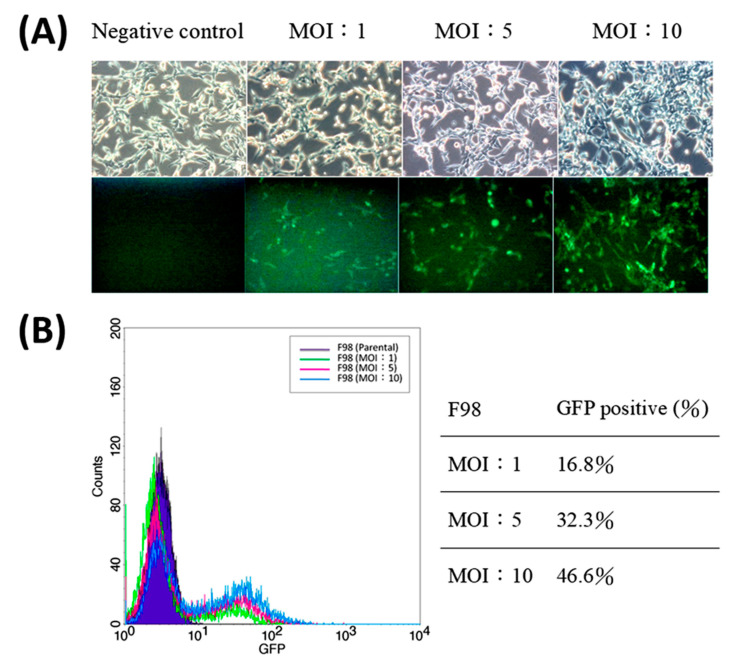
Characteristics of F98 cells transfected with *FGT* triple reporter genes. (**A**) F98/*FGT* cells were observed using a fluorescent microscope (Leica DM6000B, Wetzlar, Germany) with 100× magnification under multiplicity of infection (MOIs) of 1, 5, and 10 (*FGT*: F, firefly luciferase; G, enhanced green fluorescent protein; and T, thymidine kinase). (**B**) The intensities of green fluorescence were analyzed with a FACScan flow cytometer (Beckman Coulter CytoFLEX, Brea, CA, USA). The percentages of GFP-positive F98/*FGT* cells were 16.8%, 32.3%, and 46.6% for MOI-1, -5, and -10, respectively.

The transfected F98 cells were observed by a fluoroscopy microscope, and the MOI-1, -5, and -10 (multiplicity of infection) patching conditions did not cause any significant change of cell morphology (Figure 3A). It can be seen that as the virus concentration is higher, the brightness of green fluorescent light and the proportion of bright cells are higher. The ratio of green fluorescence is quantified by flow cytometry, showing the proportions of GFP-positive cells for MOI-1, -5, and -10 as 16.8%, 32.3%, and 46.6%, respectively (Figure 3B).

### 2.4. Screening and Sorting of High Purity of F98/FGT Cells with GFP Reporter

**Figure 4:**
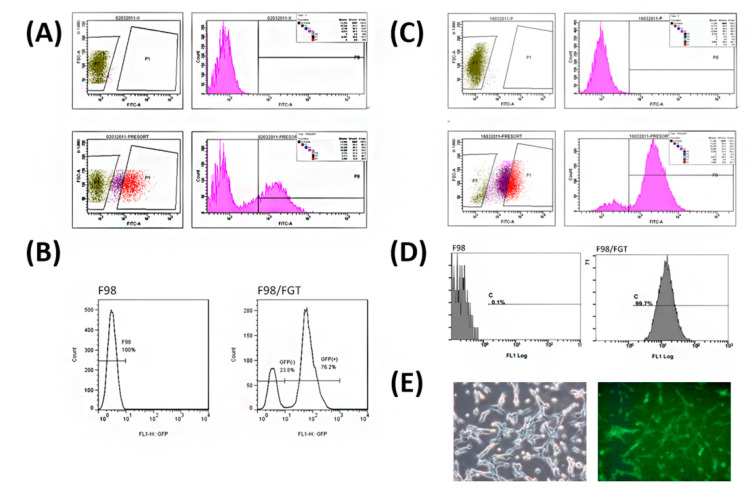
Further sorting of MOI-10 F98/*FGT* cells using green fluorescent protein (GFP) reporter and FACScan flow cytometer (FCM) for selection. (**A**) Top left: parental F98 cells were used as the negative control to provide two regions using 2 × 10^5^ as the cut-off point—P7 for parental F98 cells and P1 for high intensity of green fluorescent F98/*FGT* cells. Top right: the green fluorescent intensity (i.e., FITC-A) of F98 parental cells was all less than 2 × 10^2^. Bottom left showed the P7 and P1 for F98/*FGT* cells. Bottom right: after four subcultures of F98/*FGT* cells from P1 area, those cells in P8 region (the green fluorescent intensity > 5 × 10^2^) were sorted and analyzed with FCM. (**B**) Flow cytometer analysis showed that 100% of parental F98 cells (left) were GFP(−), and 76.2% of F98/*FGT* cells (right) were GFP(+). (**C**) The selection protocol as shown in (A) and (B) were repeated to obtain higher percentage of GFP(+) F98/*FGT* cells. (**D**) Flow cytometric analysis showed that 99.7% of F98/*FGT* cells were GFP(+). (**E**) High percentage GFP(+) F98/*FGT* cells were photographed under fluorescent microscope, and it was confirmed that >90% cells were GFP(+) (100× magnification).

The F98/*FGT* cells could be further purified and amplified for the use of the animal study. Both the cell size and the fluorescence intensity of parental F98 cells were used as the criteria for the negative control. The cells with the highest transfection efficiency of MOI-10 were used for screening the F98/*FGT* cells, and those cells with fluorescence intensity of more than 2 × 10^5^ (shown as P1 in the bottom left panel of Figure 4A) were about 26% of all cells, which were further subcultured four generations and then analyzed with FCM. The F98/*FGT* cells with the green fluorescent intensity >5 × 10^2^ were designated as P8 (shown in bottom right panel of Figure 4A) and were further subcultured four times and then analyzed with FCM. About 76.2% GFP(+) F98/*FGT* cells were obtained (Figure 4B, right panel). The above protocol was repeated second time again, and finally, 99.7% GFP(+) F98/*FGT* cells were obtained as shown in Figure 4C,D. The secondary sorted GFP(+) F98/*FGT* cells were randomly sampled and examined under fluorescent microscope and found that more than 90% of F98/*FGT* cells were GFP positive (Figure 4E), which were further used for the animal experiments.

### 2.5. Using Luciferase Activity for Monitoring the Cell Growth of F98/FGT Cells

**Figure 5:**
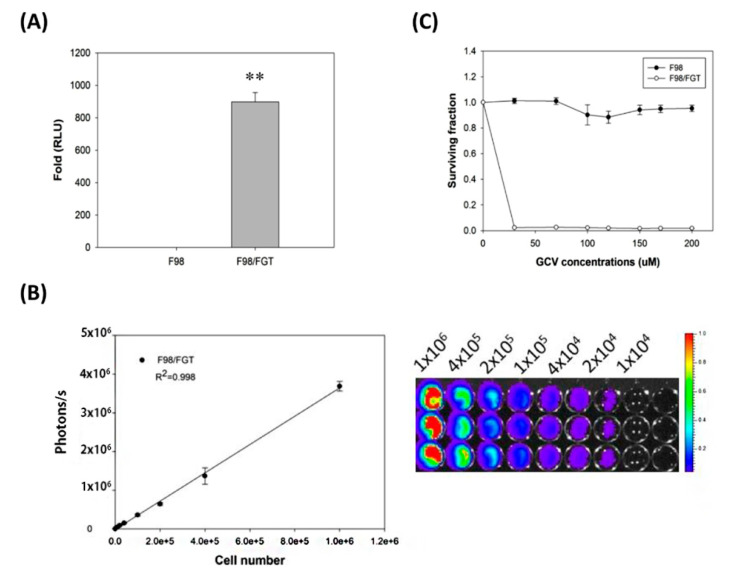
Validation of constructed luciferase and thymidine kinase (*tk*) genes and their expressions. (**A**) The expressions of luciferase gene of F98 and F98/*FGT* cells were presented as relative luminometer units (RLU). The difference of RLU between two cell lines were about 900 times and was significant (** *p* < 0.01). (**B**) The luciferase activity (photons/second) of F98/*FGT* glioma was proportional to the cell number with *R*^2^ = 0.998. (**C**) Ganciclovir (GCV) and MTT assay were used to validate the *tk* expression. About 99% of F98/FGT cells were killed when concentration of GCV ≧ 30 µM, while F98 parental cells were not affected.

The luminescence signal produced by luciferase activity was established in a quantitative manner. First, it is capable to distinguish the real signal from the background. The results showed that the photon flux released per unit protein of F98 and F98/*FGT* was measured and the fold difference was as high as 897.67 (Figure 5A). Second, the number of cells is highly linearly related to the number of photons (*R*^2^ = 0.998). It is calculated that each cell emits about 3.5 photons per second (Figure 5B).

### 2.6. Thymidine Kinase Activity Distinguishes F98/FGT from F98 after Ganciclovir Treatment

The F98/*FGT* tumor growth were very sensitive to the treatments of ganciclovir above 30 μM with the overall surviving fraction of F98/*FGT* cells less than 10% (Figure 5C). Comparatively, the survival rates of F98 control group were not sensitive to the ganciclovir (GCV) treatment (Figure 5C).

### 2.7. Noninvasive Luminescence and MRI Images Successfully Track the Tumor Growth, and Curcumin indeed Acts as a Radiosensitizer

**Figure 6:**
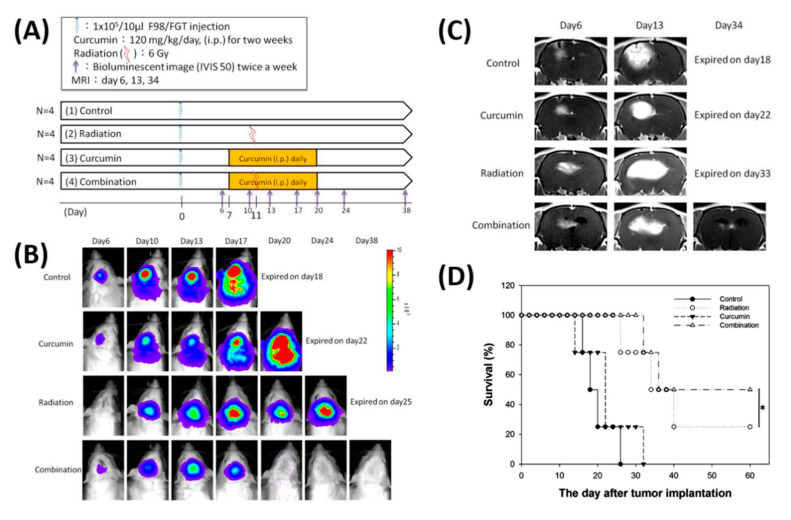
Therapeutic efficacy evaluation of curcumin, radiation, and combination of both in F98/*FGT* glioblastoma-bearing rat model using multimodalities of molecular imaging. (**A**) The experimental design and protocol. (**B**) The tumor growth inhibition was monitored by bioluminescent imaging (BLI) using Xenogen IVIS-50 system. No BLI was found on day 20 and thereafter in combination group. (**C**) The tumor growth inhibition was monitored with a 3T MRI instrument. (**D**) The Kaplan–Meier survival curves of animal study. The survival periods were 26 days and 32 days for the control and curcumin alone, respectively. About one-fifth and half of rats survived more than 60 days in the radiation group and combination group, respectively. Notably, a significance in survival was found between these two groups (* *p* < 0.05). The experiments were repeated twice and one out of two is shown here.

The design for examining whether curcumin can serve as a radiosensitizer is shown in Figure 6A, and it can be observed that the enhancement of luminescent signal fits well with the growth of brain tumors in the control group (Figure 6B). The inhibitory effect of curcumin alone on tumors was obvious at the early stage, and the signal decreased slightly during the 6th to 13th days. But after the 13th day, the tumor regains its growing capacity. After 1 week of radiation exposure, the radiation group showed a significant decrease in the signal, but the rats with no complete control of the tumor relapsed rapidly after the 20th day. The curcumin plus radiation group can effectively suppress the tumor growth since the signal disappeared after the combined treatment, and no signal was observed after 1-month treatment (Figure 6B). The MRI images also support the highlight findings in Figure 6B that the efficacy of combinative treatment of curcumin plus radiation is much better than that of curcumin alone or radiation alone (Figure 6C). Physiologically, as the tumor grows, the rats may suffer from extremely high brain pressure with a bleeding in the eyes and nose, delayed actions, and eating difficulties, while a weight loss of more than 20% is correlated to their final death (data not shown). From the observation of overall survival rates, 50% of the rats in the combined treatment group survived and were in good health at the end of the experiment. It has survived twice as long as the control group, and significantly better than those of curcumin alone and radiation alone groups (Figure 6D).

## 3. Discussion

GBM is characteristic of the most frequent, aggressive, high-grade, high lethal, and low-survival rate brain tumor. After the removal of maximal tumor parts with surgery, the therapeutic strategy of GBM much depends on radiation together with concurrent chemotherapy. The criteria of not only causing no or least damage to the normal brain cells but also conquering the difficulty of passing blood–brain barrier urged the clinical and translational scientists to figure out potential radiosensitizers, which should be examined in preclinical animal models, especially those noninvasive and dynamically trackable ones.

The rapid developments of medical imaging technologies greatly help scientists to establish GBM animal models that can sensitively detect the expression of reporter genes. In 2008, Bryant and his colleagues firstly established a brain tumor model in rats bearing F98 cells carrying *luc* gene. In the pilot rat model, the growth of GBM was successfully followed up with correlated luminescence signal in which the correlation efficiency was about 0.85 [29]. Following their example, in 2014, our team had successfully extended the applications of GBM animal model to a double-reporter gene F98/*tk-luc* tumor-bearing rat model, whose bioluminescence signals can be accurately detected with multimodality imaging instruments [30]. In that module, the growth of in situ brain tumor was visible and quantitatively followed up with the expressions of *luc* and *HSV1-tk*, enlightening the access to examine the feasibility of candidates to serve as GBM drugs and/or radiosensitizers. However, the efficiency of successful transfection and success of carrying the double-reporter gene F98/*tk-luc* among the investigated GBM cells are still not satisfying. As mentioned, the transfection efficiency in highest MOI group is less than 50% (Figure 3).

In the current study, we constructed the *pFu-FGT* lentivirus with three reporter genes, transfected it into the glioma F98 cell line again, and evaluated the capacities of curcumin to serve as a radiosensitizer for GBM therapeutic practice. In the most updated triple reporter gene lentivirus design, *GFP*, whose expression is far from detectible in rats, has greatly strengthened the convenience of checking the carrying and expression of triple reporter gene lentivirus among GBM cell population with simple observation under fluorescent microscope. In one word, it plays critically in sorting the GBM cells at the bridging step from cell observations into animal models.

Curcumin, a strong antioxidant and anti-inflammatory agent, has been moved from kitchen to bench examining of its anticancer capacities in aspects of inhibition of cancer initiation, progression, and metastasis [21,31]. In addition to a panel of cancers [21,25,32,33], curcumin is also capable in inhibiting the growth of glioma and glioblastoma cells [34,35]. In literature, curcumin can induce GBM cells to die in forms of autophagy [36] and apoptosis [37]. Also, curcumin is capable to enhance the cytotoxicity of temozolomide in GBM cells [36]. Our results concluded that, similar to those reported in human glioma U251 cells [38,39], curcumin can cause G2/M arrest in glioma F98 cells and increase the sensitivity of glioma cells to radiation (Figure 2). 

In recent years, curcumin has been reported to serve as potential radiosensitizer in peripheral blood lymphocytes, breast, and prostate cancer cells [40,41,42], but never in GBM cells. To bridge the feasibility of curcumin as radiosensitizer from cell examination to clinical practice, the experiments in GBM animal models are critical and essential. First, in GMB tumor cell-transplanted rats, the treatment of curcumin alone has been reported to slow down the growth of GBM tumors and prolong the survival periods of GBM-bearing rats [24,43]. Second, as for those in situ GBM animals, it is excitingly confirmed that curcumin can pass through the blood–brain barrier and makes no damage to normal brain tissue [44]. Third, curcumin itself is of extremely low toxicity to the rats, while up to 5 g/kg dosage of curcumin causes almost no side effect in rats [45]. Forth, daily oral dose tolerance can be as high as 12 g without any side effect in the first phase of clinical trial [46].

The suffering of GBM patients from general recurrence after radiation therapy and extremely low survival rates after recurrence have urged the clinical and translational scientists to figure out more practical radiosensitizer(s) [47]. The temporary successfulness of curcumin should not stop our trails in each step from bench to bed. There are still some challenges such as curcumin is rapidly digested and metabolized in the body, and the ability of the body to absorb curcumin and its bioavailability are also problems to be considered. Also, the methodology for effective accumulation of curcumin in a wide range of brain tumors is still under development. Further efforts to translate the current preclinical knowledge to the real application of curcumin in combinatorial radiotherapeutic strategies in clinical settings are necessary.

In conclusion, we demonstrated that curcumin could be combined with a novel triple-reporter F98/FGT glioma-bearing rat model for the therapeutic efficacy evaluation of GBM therapy. This system may also be applied for screening the potential GBM drugs and radiosensitizers. In addition, a noninvasive and real-time multimodalities of imaging system could effectively shorten this screening processes.

## 4. Materials and Methods

### 4.1. Cell Culture

F98 glioblastoma cells were cultured in Dulbecco’s modified eagle medium (DMEM, cat. no. 12100-046, Gibco, Grand Island, NY, USA) containing 10% fetal bovine serum (FBS, Hyclone, Thermo Scientific, Rockford, IL, USA) and 1% penicillin–streptomycin solution (Gibco, Grand Island, NY, USA) in an incubator at 37 °C and was supplied with 5% CO_2_. 

### 4.2. Transfection and Selection of F98 with pFu-FGT Lentivirus

Briefly, 3 × 10^6^ F98 cells were incubated in 1 mL OptiMEM plus 8 μg/mL polybrene with the *pFu-FGT* lentivirus (F stands for *Fluc*: firefly luciferase; G stands for *eGFP*: enhanced green fluorescent protein; and T stands for *tk*: thymidine kinase at concentrations of multiplicity of infection (MOI) 1, MOI 5, and MOI 10, respectively; Figure 1A). The cells were cultured in T75 flasks for 4 h, then directly added with DMEM medium (cat. no. 12100-046, Gibco, Grand Island, NY, USA). After 24 h, the virus-containing culture medium was replaced with the fresh culture medium, and cells were continuously subcultured four times. Those F98 cells transfected with *pFu-FGT* vector at MOI 10 were prepared in a cell density of 10^7^ cells/mL phosphate buffer (PBS) and sorted with a FACScan flow cytometer (FCM, Beckman Coulter CytoFLEX, Brea, CA, USA). Once about 80% of transfected cells were GFP(+), judged by observing with a fluorescent microscope (Leica DM6000B, Wetzlar, Germany), these F98/*FGT* cells were further purified and amplified according to the particle size and fluorescent intensity by setting the cut-off point of the green fluorescent intensity at 5 × 10^2^ using parental F98 cells as the negative control, which was designated as P7. The F98/*FGT* cells with the green fluorescent intensity >5 × 10^2^ were designated as P1. Then, only 30% of P1 cells with higher fluorescent intensity were sorted and designated as P8 and were further subcultured another four times followed by FCM analysis. The above selection protocol was repeated second time until >90% of F98/*FGT* cells were GFP(+). These cells were used for the animal experiments.

### 4.3. Assay of Luciferase Activity of F98/FGT Cells

F98 and F98/FGT cells were planted in a 6-well culture dish. After the cells were full, the cell culture medium was removed, and 200 μL of 1× lysis buffer was added to each well after washing with PBS. Then the cells were thawed in a refrigerator at −80 °C for 10 min. The cells in the wells were scraped off and the cell fluid was collected and centrifuged at 4 °C (>12,000 rpm for 2 min). Then, 30 μL of the supernatant was aspirated into a black 96-well plate, 100 μL of luciferin buffer was added to each well, and the measured photon number was divided from the protein concentration. In a black 96-well plate, 1 × 10^6^, 4 × 10^5^, 2 × 10^5^, 1 × 10^5^, 4 × 10^4^, 2 × 10^4^, and 1 × 10^4^ F98/*FGT* cells were planted in each well, with three replicates for each number. Precisely, 500 μM of D-luciferin was added to each well, and after standing for 5 min, imaging was performed using the IVIS-50 system (Xenogen, Alameda, CA, USA) for 5 min. Using the Living Image software (version 2.20, Xenogen) to circle the regions-of-interest (ROIs) to quantify the number of photons. The correlation between the number of cells and the number of photons was calculated and the average number of photons emitted per second per cell was also estimated.

### 4.4. Assay of Thymidine Kinase Activity

Precisely, 3 × 10^3^ F98 or F98/*FGT* cells were separately seeded in 96-well culture plates. After 24 h, when the cells were all attached to the plates, each well was added with various concentrations (0–200 μM) of ganciclovir (GCV). Each concentration of GCV is repeated for at least six times. The cells were incubated in 96-well plates for 5 days. Then, the cell culture medium containing GCV was removed, and after adding 1 mg/mL of 3-(4,5-dimethylthiazol-2-yl)-2,5-diphenyltetrazolium bromide (MTT, Sigma-Aldrich, St. Louis, MO, USA) solution, it was placed in an incubator for 4 h. Then, the formation of purple crystals was observed. Further, the MTT solution was removed, 100 μL of dimethyl sulfoxide (DMSO, D2650, Sigma-Aldrich) was added to dissolve the purple crystals, and an ELISA reader (Power Wave X340; Bio-Tek Instrument, Inc., Winooski, VT, USA) was used to detect the absorbance at 570 nm. 

### 4.5. Viability of Curcumin-Treated Cells

F98 cells were seeded overnight in 96-well plates (3 × 10^4^ cells per well). Then, various concentrations (0, 5, 10, 20, 40, 80, and 160 μM) of curcumin were added to them and the plates were cultured for another 24 h. In the culture medium containing curcumin, MTT solution was added at a final concentration of 0.5 mg/mL (diluted 5 mg/mL MTT stock solution using serum-free medium), and the plates were placed in the incubator for 4 h to allow the formation of purple crystals. The MTT solution was then removed, 100–200 μL of dimethyl sulfoxide (DMSO) was added to dissolve the purple crystals, and an ELISA reader (Power Wave X340; Bio-Tek Instrument, Inc., Winooski, VT, USA) was used to detect the absorbed light at a wavelength of 570 nm. The analytical readings were calculated as the ratio of the curcumin-treated group to the control group, which was set at 100%. 

### 4.6. Cell Cycle Analysis after Curcumin Treatment

In brief, 7 × 10^5^ F98 cells/dish were planted in 6 cm diameter dishes. The next day, different concentrations (0, 5, 10, 15, and 20 μM) of curcumin were added. After 24 h of treatment, the cells and the culture solution were collected together, centrifuged at 3500 rpm for 10 min, and the cells were dispersed, washed with PBS, and centrifuged again. The cells were fixed with 70% alcohol and placed overnight at −20 °C. The procedure for cell washing with the same PBS was repeated twice, and the cells were stained with propidium iodide for 30 min. After sieving, the cells were analyzed by flow cytometry.

### 4.7. Colony Formation Assay for Cells Irradiated with Radiation Alone and Combined with Curcumin

In particular, 7 × 10^5^ F98 cells were cultured overnight in T25 flask. Then, 15 μM curcumin was added (this step was ignored in the radiation only group) and kept for another 24 h. Cells in these flasks were combined with radiation of 0, 1, 2, 4, 8, and 12 Gy with X-ray biological irradiator (RS 2000; Rad Source Technologies, Suwanee, GA, USA), and the following parameters were maintained: 1.03 Gy/min, 80 cm source-to-surface distance (SSD), and field size 30 × 30 cm^2^. After 14 days of culture, the cells were fixed with a 3:1 methanol-glacial acetic acid for 10 min, and then rinsed with tap water. Cell staining was performed by adding 2% crystal violet for 10 min. Then, the cells were rinsed with tap water and air dried. Those colonies with more than 50 cells were counted as survival ones. The control group that received neither radiation exposure nor curcumin treatment was used as the plating efficiency. The cell surviving fraction was calculated by the following formula:

$$\mathrm{Surviving}\;\mathrm{Fraction}=\frac{\mathrm{No}.\;\mathrm{of}\;\mathrm{counted}\;\mathrm{colony}}{\mathrm{No}.\;\mathrm{of}\;\mathrm{cells}\;\mathrm{seeded}\times \left(\mathrm{Plating}\;\mathrm{efficiency}/100\right)}$$

### 4.8. F98/FGT Animal Model

All the animal experiments were approved by the IACUC of National Yang-Ming University with the protocol number: 1071107. Eight- to 10-week old male Fischer (F344/NNarl) rats weighing about 220–280 g were purchased from National Animal Center, Taipei, Taiwan. Before operation with the brain tumor implantation, the rats were anesthetized by intraperitoneal injection with 50 mg/kg pentobarbital. After the rats were comatose, the head hairs were shaved and fixed with a stereo positioner. The povidone-iodine solution was used to disinfect the head skin of the rat, and at the intersection of the center of the two ears at the intersection of the nose, a few centimeters longitudinal cut was given. After scraping the periosteum on the rat skull, the lambda was used as a center, 3 mm to the left and 5 mm to the front, and a drill with a width of 1 mm was used to dig a hole in the skull. The F98/*FGT* cells (1 × 10^5^ cells/10 µL) were dissolved in HBSS (Gibco, cat. no. 14175), without calcium and magnesium, and placed on ice. Then, the cells were injected with a microinjection needle at a depth of 5 mm in the left brain. The microinjection needle was retained for about 5 min before being removed. The small holes in the rat skull were sealed with bone wax, and the rat scalp wound was clamped with a clip; the clip was removed after the wound was healed.

### 4.9. Treatment Strategy and Experimental Design Flow Chart

Rats bearing F98/*FGT* tumors were divided into four groups. Control group: 2 mL/kg/day corn oil was intraperitoneally injected on the 7th to 20th day after the tumor was implanted. Curcumin group: rats were intraperitoneally injected with 120 mg/2 mL/kg/day of curcumin on the 7th to 20th day. Radiotherapy group: on the 11th day after the tumor was implanted, the rats were given a single dose of 6 Gy of radiation whole brain irradiation, and the body part was given 8 mm lead protection. In the combined treatment group, the rats were intraperitoneally injected with 120 mg/2 mL/kg/day of curcumin on the 7th to 20th day, and the whole brain was irradiated with 6 Gy on the 11th day, and the rats were also given 8 mm lead protection. During the treatment period, the tumor growth changes were observed by bioluminescence and magnetic resonance imaging, and the body weight and survival time were also tracked.

### 4.10. Noninvasive Luminescence Imaging for Tracking the Tumor Growth

Rats were given 2–3% isoflurane for anesthesia, and then they were intraperitoneally injected with 150 mg/kg of D-luciferin. After 10 min, images were collected using the IVIS 50 system for 5–10 min. The regions-of-interest (ROIs) were circled and the numbers of photons were quantified with Living Image (R) software version 2.20.1. The tumor growth was monitored twice a week.

### 4.11. MRI System for Tracking the Tumor Growth in Brain

After the rats were anesthetized, 0.2 mmol/kg of gadolinium contrast agent (Gadavist) was injected from the tail vein, and 5 min after the injection, the rats were studied under 3T MRI instrument (TRIO, 3-T, Siemens MAGNETIOM, Germany). The contrast condition for T1W image were as follows: repetition time (TR) = 435 ms, echo time (TE) = 12 ms, matrix: 154 × 256, field -of-view (FOV) = 37 × 50 mm^2^, slice = 1.5 mm. At the time of angiography, the rats’ heads were fixed in a toroidal coil with a diameter of about 4 cm.

### 4.12. Statistics

All the results were presented as mean ± standard deviation (S.D.). The statistics of cell cycle and bioluminescent activity were analyzed by Student’s *t*-test for the comparison between the control and experimental groups. Animal study results were performed using one-way ANOVA followed by Tukey’s post hoc test. Differences between the means were considered significant if *p* < 0.05 (* *p* < 0.05, ** *p* < 0.01, and *** *p* < 0.001).

## References

[1] Goradel N.H., Ghiyami-Hour F., Jahangiri S., Negahdari B., Sahebkar A., Masoudifar A., Mirzaei H. (2018). Nanoparticles as new tools for inhibition of cancer angiogenesis. J. Cell Physiol..

[2] Saadatpour Z., Bjorklund G., Chirumbolo S., Alimohammadi M., Ehsani H., Ebrahiminejad H., Pourghadamyari H., Baghaei B., Mirzaei H.R., Sahebkar A. (2016). Molecular imaging and cancer gene therapy. Cancer Gene Ther..

[3] Mirzaei H., Sahebkar A., Sichani L.S., Moridikia A., Nazari S., Sadri Nahand J., Salehi H., Stenvang J., Masoudifar A., Mirzaei H.R. (2018). Therapeutic application of multipotent stem cells. J. Cell Physiol..

[4] Mirzaei H.R., Sahebkar A., Salehi R., Nahand J.S., Karimi E., Jaafari M.R., Mirzaei H. (2016). Boron neutron capture therapy: Moving toward targeted cancer therapy. J. Cancer Res. Ther..

[5] Mirzaei H.R., Mirzaei H., Lee S.Y., Hadjati J., Till B.G. (2016). Prospects for chimeric antigen receptor (CAR) gammadelta T cells: A potential game changer for adoptive T cell cancer immunotherapy. Cancer Lett..

[6] Shahar T., Nossek E., Steinberg D.M., Rozovski U., Blumenthal D.T., Bokstein F., Sitt R., Freedman S., Corn B.W., Kanner A.A. (2012). The impact of enrollment in clinical trials on survival of patients with glioblastoma. J. Clin. Neurosci..

[7] Park J.K., Hodges T., Arko L., Shen M., Dello Iacono D., McNabb A., Olsen Bailey N., Kreisl T.N., Iwamoto F.M., Sul J. (2010). Scale to predict survival after surgery for recurrent glioblastoma multiforme. J. Clin. Oncol..

[8] Senders J.T., Staples P., Mehrtash A., Cote D.J., Taphoorn M.J.B., Reardon D.A., Gormley W.B., Smith T.R., Broekman M.L., Arnaout O. (2020). An Online Calculator for the Prediction of Survival in Glioblastoma Patients Using Classical Statistics and Machine Learning. Neurosurgery.

[9] Jeon H.Y., Park C.G., Ham S.W., Choi S.H., Lee S.Y., Kim J.Y., Seo S., Jin X., Kim J.K., Eun K. (2017). BRM270, a Compound from Natural Plant Extracts, Inhibits Glioblastoma Stem Cell Properties and Glioblastoma Recurrence. J. Med. Food.

[10] Barani I.J., Larson D.A. (2015). Radiation therapy of glioblastoma. Cancer Treat. Res..

[11] Storey K., Leder K., Hawkins-Daarud A., Swanson K., Ahmed A.U., Rockne R.C., Foo J. (2019). Glioblastoma Recurrence and the Role of O(6)-Methylguanine-DNA Methyltransferase Promoter Methylation. JCO Clin. Cancer Inform..

[12] Monteiro A.R., Hill R., Pilkington G.J., Madureira P.A. (2017). The Role of Hypoxia in Glioblastoma Invasion. Cells.

[13] Zanders E.D., Svensson F., Bailey D.S. (2019). Therapy for glioblastoma: Is it working?. Drug Discov. Today.

[14] Alifieris C., Trafalis D.T. (2015). Glioblastoma multiforme: Pathogenesis and treatment. Pharmacol. Ther..

[15] Garnier D., Meehan B., Kislinger T., Daniel P., Sinha A., Abdulkarim B., Nakano I., Rak J. (2018). Divergent evolution of temozolomide resistance in glioblastoma stem cells is reflected in extracellular vesicles and coupled with radiosensitization. Neuro. Oncol..

[16] Aggarwal B.B., Sundaram C., Malani N., Ichikawa H. (2007). Curcumin: The Indian solid gold. Adv. Exp. Med. Biol..

[17] Nelson K.M., Dahlin J.L., Bisson J., Graham J., Pauli G.F., Walters M.A. (2017). The Essential Medicinal Chemistry of Curcumin. J. Med. Chem..

[18] Menon V.P., Sudheer A.R. (2007). Antioxidant and anti-inflammatory properties of curcumin. Adv. Exp. Med. Biol..

[19] Srivastava G., Mehta J.L. (2009). Currying the heart: Curcumin and cardioprotection. J. Cardiovasc. Pharmacol. Ther..

[20] Mythri R.B., Bharath M.M. (2012). Curcumin: A potential neuroprotective agent in Parkinson’s disease. Curr. Pharm. Des..

[21] Aggarwal B.B., Kumar A., Bharti A.C. (2003). Anticancer potential of curcumin: Preclinical and clinical studies. Anticancer Res..

[22] Ahmed T., Gilani A.H. (2014). Therapeutic potential of turmeric in Alzheimer’s disease: Curcumin or curcuminoids?. Phytother. Res..

[23] Pan J., Li H., Ma J.F., Tan Y.Y., Xiao Q., Ding J.Q., Chen S.D. (2012). Curcumin inhibition of JNKs prevents dopaminergic neuronal loss in a mouse model of Parkinson’s disease through suppressing mitochondria dysfunction. Transl. Neurodegener..

[24] Perry M.C., Demeule M., Regina A., Moumdjian R., Beliveau R. (2010). Curcumin inhibits tumor growth and angiogenesis in glioblastoma xenografts. Mol. Nutr. Food Res..

[25] Hsiao Y.T., Kuo C.L., Chueh F.S., Liu K.C., Bau D.T., Chung J.G. (2018). Curcuminoids Induce Reactive Oxygen Species and Autophagy to Enhance Apoptosis in Human Oral Cancer Cells. Am. J. Chin. Med..

[26] Li M., Wang Y., Liu M., Lan X. (2018). Multimodality reporter gene imaging: Construction strategies and application. Theranostics.

[27] Youn H., Chung J.K. (2013). Reporter gene imaging. AJR Am. J. Roentgenol..

[28] Yang C., Tian R., Liu T., Liu G. (2016). MRI Reporter Genes for Noninvasive Molecular Imaging. Molecules.

[29] Bryant M.J., Chuah T.L., Luff J., Lavin M.F., Walker D.G. (2008). A novel rat model for glioblastoma multiforme using a bioluminescent F98 cell line. J. Clin. Neurosci..

[30] Chien Y.C., Chen J.C., Lin W.C., Ding H.J., Wang H.E., Kao C.H., Hwang J.J. (2014). Using [(1)(8)F]FBAU for imaging brain tumor progression in an F98/tk-luc glioma-bearing rat model. Oncol. Rep..

[31] Edwards R.L., Luis P.B., Varuzza P.V., Joseph A.I., Presley S.H., Chaturvedi R., Schneider C. (2017). The anti-inflammatory activity of curcumin is mediated by its oxidative metabolites. J. Biol. Chem..

[32] Falke J., Parkkinen J., Vaahtera L., Hulsbergen-van de Kaa C.A., Oosterwijk E., Witjes J.A. (2018). Curcumin as Treatment for Bladder Cancer: A Preclinical Study of Cyclodextrin-Curcumin Complex and BCG as Intravesical Treatment in an Orthotopic Bladder Cancer Rat Model. Biomed. Res. Int..

[33] Selvam C., Prabu S.L., Jordan B.C., Purushothaman Y., Umamaheswari A., Hosseini Zare M.S., Thilagavathi R. (2019). Molecular mechanisms of curcumin and its analogs in colon cancer prevention and treatment. Life Sci..

[34] Ambegaokar S.S., Wu L., Alamshahi K., Lau J., Jazayeri L., Chan S., Khanna P., Hsieh E., Timiras P.S. (2003). Curcumin inhibits dose-dependently and time-dependently neuroglial cell proliferation and growth. Neuro. Endocrinol. Lett..

[35] Park K.S., Yoon S.Y., Park S.H., Hwang J.H. (2019). Anti-Migration and Anti-Invasion Effects of Curcumin via Suppression of Fascin Expression in Glioblastoma Cells. Brain Tumor Res. Treat..

[36] Zanotto-Filho A., Braganhol E., Klafke K., Figueiro F., Terra S.R., Paludo F.J., Morrone M., Bristot I.J., Battastini A.M., Forcelini C.M. (2015). Autophagy inhibition improves the efficacy of curcumin/temozolomide combination therapy in glioblastomas. Cancer Lett..

[37] Bangaru M.L., Chen S., Woodliff J., Kansra S. (2010). Curcumin (diferuloylmethane) induces apoptosis and blocks migration of human medulloblastoma cells. Anticancer Res..

[38] Wang L., Ye X., Cai X., Su J., Ma R., Yin X., Zhou X., Li H., Wang Z. (2015). Curcumin suppresses cell growth and invasion and induces apoptosis by down-regulation of Skp2 pathway in glioma cells. Oncotarget.

[39] Liu E., Wu J., Cao W., Zhang J., Liu W., Jiang X., Zhang X. (2007). Curcumin induces G2/M cell cycle arrest in a p53-dependent manner and upregulates ING4 expression in human glioma. J. Neurooncol..

[40] Minafra L., Porcino N., Bravata V., Gaglio D., Bonanomi M., Amore E., Cammarata F.P., Russo G., Militello C., Savoca G. (2019). Radiosensitizing effect of curcumin-loaded lipid nanoparticles in breast cancer cells. Sci. Rep..

[41] Sebastia N., Montoro A., Hervas D., Pantelias G., Hatzi V.I., Soriano J.M., Villaescusa J.I., Terzoudi G.I. (2014). Curcumin and trans-resveratrol exert cell cycle-dependent radioprotective or radiosensitizing effects as elucidated by the PCC and G2-assay. Mutat. Res..

[42] Chendil D., Ranga R.S., Meigooni D., Sathishkumar S., Ahmed M.M. (2004). Curcumin confers radiosensitizing effect in prostate cancer cell line PC-3. Oncogene.

[43] Erices J.I., Torres A., Niechi I., Bernales I., Quezada C. (2018). Current natural therapies in the treatment against glioblastoma. Phytother. Res..

[44] Purkayastha S., Berliner A., Fernando S.S., Ranasinghe B., Ray I., Tariq H., Banerjee P. (2009). Curcumin blocks brain tumor formation. Brain Res..

[45] Aoki H., Takada Y., Kondo S., Sawaya R., Aggarwal B.B., Kondo Y. (2007). Evidence that curcumin suppresses the growth of malignant gliomas in vitro and in vivo through induction of autophagy: Role of Akt and extracellular signal-regulated kinase signaling pathways. Mol. Pharmacol..

[46] Sharma R.A., Euden S.A., Platton S.L., Cooke D.N., Shafayat A., Hewitt H.R., Marczylo T.H., Morgan B., Hemingway D., Plummer S.M. (2004). Phase I clinical trial of oral curcumin: Biomarkers of systemic activity and compliance. Clin. Cancer Res..

[47] Caretti V., Zondervan I., Meijer D.H., Idema S., Vos W., Hamans B., Bugiani M., Hulleman E., Wesseling P., Vandertop W.P. (2011). Monitoring of tumor growth and post-irradiation recurrence in a diffuse intrinsic pontine glioma mouse model. Brain Pathol..

